# 3D printing in medical imaging and healthcare services

**DOI:** 10.1002/jmrs.292

**Published:** 2018-07-03

**Authors:** Kamarul A. Abdullah, Warren Reed

**Affiliations:** ^1^ Discipline of Medical Radiation Sciences Faculty of Health Sciences The University of Sydney Lidcombe New South Wales Australia; ^2^ Faculty of Health Sciences Universiti Sultan Zainal Abidin Terengganu Malaysia

## Abstract

Three‐dimensional (3D) printing technology has demonstrated a huge potential for the future of medicine. Since its introduction, it has been used in various areas, for example building anatomical models, personalising medical devices and implants, aiding in precision medical interventions and the latest development, 3D bioprinting. This commentary is provided to outline the current use of 3D printing in medical imaging and its future directions for advancing the healthcare services.

Medical imaging is at the core of the healthcare industry due to its wide applications in nearly all patient‐related management. The use of computed tomography (CT), magnetic resonance imaging (MRI) and other medical imaging modalities have clearly enabled health practitioners to diagnose patients’ conditions and thus treat them more effectively. These modalities are rapidly growing, progressing from black‐and‐white to colour, from two‐dimensional (2D) to three‐dimensional (3D) and the most recently four‐dimensional (4D). This has been allied with the emergence of 3D printing which can translate these medical imaging data sets from virtual to physical models.

Building physical models using 3D printing requires three major steps. The first step is to design virtual models of the desired object. These models can be developed from scratch using computer‐aided design (CAD) software or generated from volumetric CT or MRI image data sets. After that, an improvement of the models is performed to produce error‐free files. The completed model data sets are then exported to the 3D printer to build the physical models. At the final stage, the appropriate materials and printer settings are carefully selected to produce high‐quality 3D‐printed models. A summary of these major steps can be shown in Figure 1.

**Figure 1:**
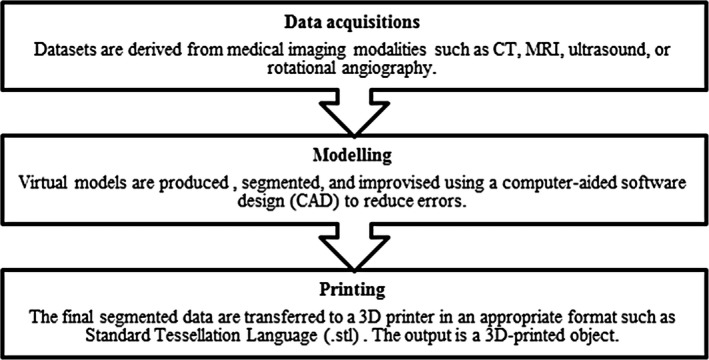
Three major steps of developing a 3D‐printed object.

An example of developing a physical 3D‐printed model using these three steps is shown in our previous work which is entitled “*Development of an organ‐specific insert phantom generated using a 3D printer for investigations of cardiac computed tomography protocols*”.^1^ We found that the resultant images, particularly attenuation values, were comparable to the image data sets of a real‐patient and Catphan^®^ 500 phantom.

In medical imaging, 3D printing is mainly used to produce various anatomical models of the human body. The physical interaction with these 3D‐printed models allows the physicians and surgeons to enhance the visualisation of lesions, planning of surgical procedures and communication with patients. However, these 3D‐printed anatomical models can only serve their purpose if there is sufficient information from the volumetric image data sets. CT and MRI scanners are most often used for producing these high information volumetric image data sets, but other modalities are also possible to use. For example, Materialise Inc., a 3D printing company, demonstrated that the volumetric image data sets from 3D ultrasound and rotational angiography could also produce similar high‐quality 3D printing models.^2^

3D printing technology is also used to create various personalised medical devices and implants that have improved the lives of many people around the world. These models are usually very patient‐specific and contain very detailed information as it is vital to ensure the 3D‐printed models produced can fit accurately in the patients’ body such as in the example of prosthetics. Two examples of pertinent cases that use 3D printing for this purpose are: (i) the case of the paediatric cardiologist, Dr Frank Ing, at Children's Hospital Los Angeles (CA, USA), who was able to modify an existing stent using a 3D‐printed model to repair a baby's artery. He reported that the 3D‐printed model had helped him to design the appropriate size during this interventional procedure.^3^; and (ii) the case of scientists at Morriston Hospital (Wales, UK) who successfully rebuilt a patient's jaw after damage caused by a tumour using a 3D‐printed titanium implant and cutting guides. They had used the volumetric CT image data sets of the unaffected side to duplicate the anatomical shape and rebuild the jaw using the 3D printer to match the area of jawbone removed.^4^

All these achievements of 3D printing technology have brought a revolutionary change in medical imaging and healthcare, and its adoption is slowly taking place in many hospitals around the world. The Ottawa Hospital is the first in Canada to open an integrated 3D printing programme that can be used for education, surgical planning and medical research. In Madrid, the team of surgeons and engineers at the Hospital General Universitario Gregorio Marañón have created a special laboratory called ‘FabLab’ which accelerates the development of 3D printing technology to the hospital. In the Sawai Man Singh Hospital (Jaipur, India), 22 surgeries have relied upon advanced 3D printing technology to aid in precision medical interventions. In the Middle East, the surgeons at Al Qassimi Hospital (Sharjah, United Arab Emirates), have been using 3D‐printed models to educate their patients on what to expect after their surgical procedures. According to the deputy CEO, Dr Saqr Al Mulla, it is the first hospital in the Middle East to use 3D printing technology for this medical purpose.^5^

Many researchers are still seeking more advanced uses of 3D printing technology. The most recent that is getting more attention worldwide is the potential to produce 3D‐printed replacement tissues and organs out of living tissue. A few companies and institutions have already initiated exploring these opportunities, and these are known as bioprinters. For instance, EnvisionTEC Inc., a company in Germany, has designed a bioprinter named the “*3D bioplotter system*”. This fabricates scaffolds utilising a range of materials from soft hydrogels over polymer melts and can also produce hard ceramics and metals. Another bioprinter is used by Organovo Holdings Inc. and has already started selling 3D‐printed liver cells and has created kidney and skin tissues. Organovo have also signed a partnership with Autodesk for the advancement of more efficient CAD software for bioprinting.

It would appear logical that the technologies used between medical imaging and 3D printing should be located in the medical imaging department at the hospital. However, it was found that the most dominant users are coming from the engineering and computer science groups who run their 3D printing laboratories themselves in in‐house hospitals. For example, the first 3D printing laboratory in‐house hospital in New South Wales has been established at Wollongong Private Hospital in their ‘Innovation Hub’.^6^ At first glance, these designated 3D printing laboratories make sense as these groups have to interact with almost every other department in a hospital to develop or design the 3D‐printed medical products. However, not all hospitals have the capabilities to establish and operate new 3D printing laboratories, and thus, some different points of view have been raised especially from other departments in the hospitals. Some of them were concerned that these designated 3D printing laboratories could impose very high operational costs and may disrupt the existing workflow. Therefore, these 3D printing laboratories could be established and operated within the existing medical imaging department in the hospitals as they also offers service across all specialities in a hospital.

The medical imaging department appears to be the most logical access point for 3D printing technology in the hospital workflow. Radiologists and radiographers have access to all of the images in the system, and they have the best understanding of images in all planes. They are already educated to be able to identify various structures and differentiate them from each other. However, experts from engineering or computer science groups could also be used to monitor and ensure the efficiency of 3D printing services. As 3D printing is likely to emerge rapidly in the medical imaging department, additional knowledge and mastery of new technical skills to generate diagnostically acceptable 3D‐printed models must be developed and could constitute an advanced or extended role for the radiographers. Early adopter radiographic staff must invest in honing these skills as later they could well will be incorporated into the normal medical imaging workflow, facilitating a new pathway to using 3D printing as a further step to improve patient care.

When a new technology has been introduced in the market, the question about how 3D printing medical products could be monitored will persist, potentially holding the technology back from universal adoption. However, the good news is that recently, the U.S. Food and Drug Administration (FDA) have just released guidance on the 3D printing of medical products.^7^ The guidance details the organisation's position on the device configuration, testing, and quality framework requirements. This guidance shows that 3D printing has a wide range of clinical applications and will help manufacturers to bring 3D‐printed models to the market more efficiently. As indicated by the FDA, the subsequent stage is to develop guidance on 3D printing that explores the role of non‐traditional manufacturing facilities, such as, hospital and university laboratories and to review the regulatory issues attached to the bioprinting of biological, cellular and tissue‐based products.

In summary, the emerging 3D printing technology in medical imaging has a lot of benefits not only to health professionals but also to their patients. The future should be to develop the advanced practice or extended role for radiographers using this technology and to fully explore how it may transform the future of medical imaging.

## Conflict of Interest

The authors declare no conflict of interest.

## References

[1] Abdullah KA , McEntee MF , Reed W , Kench PL . Development of an organ‐specific insert phantom generated using a 3D printer for investigations of cardiac computed tomography protocols. J Med Radiat Sci 2018; doi: 10.1002/jmrs.279 PMC6119733 29707915

[2] Materialise Inc. Discover the transformative potential of 3D Printing, 2018 [cited 2018 March 03]. Available from: http://www.materialise.com/en.

[3] Mannan S . 3D printed model used to produce custom stent for 18 month old with pulmonary atresia, 2017 [cited 2017 December 05]. Available from: https://www.3dmednet.com/users/19381-sonia-mannan/posts/15044-3d-printed-model-used-to-produce-custom-stent-for-18-month-old-with-pulmonary-atresia.

[4] Leask F . Swansea surgeons rebuild jaw with 3D printed implant and guides in world first, 2017 [cited 2017 December 05]. Available from: https://www.3dmednet.com/channels/332-news-views/posts/21618-swansea-surgeons-rebuild-jaw-with-3d-printed-implant-and-guides-in-world-first.

[5] Scott C , Mendoza HR , Saunders S . 3D printing in hospitals, 2017 [cited 2017 December 06]. Available from: https://3dprint.com/tag/3d‐printing‐in‐hospitals/.

[6] ARC Centre of Excellence for Electromaterials Science . Wollongong's in‐hospital 3D printing lab is a first for NSW, 2016 [cited 2017 December 12]. Available from: http://www.electromaterials.edu.au/news/wollongong-s-in-hospital-3d-printing-lab-is-a-first-for-nsw/.

[7] US Food and Drug Administration . Statement by FDA Commissioner Scott Gottlieb, M.D., on FDA ushering in new era of 3D printing of medical products; provides guidance to manufacturers of medical devices [press release], 2017 [cited 2018 May 01]. Available from: https://www.fda.gov/NewsEvents/Newsroom/PressAnnouncements/ucm587547.htm.

